# Extra-axial chordoma of the distal tibia incidentally discovered during fixation of a presumed pathologic fracture

**DOI:** 10.1093/jscr/rjag216

**Published:** 2026-04-04

**Authors:** Danil Chernov, Nicholas Frappa, Timothy Whelan, Joshua Slowinski, Matthew G Alben, Rachel Loecher, Joseph B Kuechle, Susan Daoust

**Affiliations:** Jacobs School of Medicine and Biomedical Sciences, 955 Main St. Buffalo, NY 14203, United States; Jacobs School of Medicine and Biomedical Sciences, 955 Main St. Buffalo, NY 14203, United States; Department of Orthopaedics and Sports Medicine, 462 Grider St. Buffalo, NY 14215, United States; Department of Orthopaedics and Sports Medicine, 462 Grider St. Buffalo, NY 14215, United States; Department of Orthopaedics and Sports Medicine, 462 Grider St. Buffalo, NY 14215, United States; Department of Orthopaedics and Sports Medicine, 462 Grider St. Buffalo, NY 14215, United States; Department of Orthopaedics and Sports Medicine, 462 Grider St. Buffalo, NY 14215, United States; Department of Orthopaedics and Sports Medicine, 462 Grider St. Buffalo, NY 14215, United States

**Keywords:** chordoma, extra-axial chordoma, distal tibia, pathologic fracture, orthopedic trauma, rare neoplasm

## Abstract

Chordomas are rare malignant tumors of notochordal origin that typically arise along the axial skeleton. Extra-axial chordomas involving the appendicular skeleton are exceedingly uncommon and may present diagnostic challenges due to nonspecific clinical and radiographic features. We report a case of a 59-year-old woman who sustained a low-energy distal tibial fracture and proximal fibula fracture and was found intraoperatively to have an extra-axial chordoma of the distal tibia initially presumed to be a benign cystic lesion. The tumor was excised at the time of fracture fixation, and pathologic analysis confirmed the diagnosis of extra-axial chordoma. This case highlights a highly unusual traumatic presentation of a distal appendicular chordoma and emphasizes the importance of maintaining suspicion for occult pathology in atypical cortical lesions encountered during orthopedic trauma surgery.

## Introduction

Chordomas are rare, slow-growing malignant neoplasms originating from embryologic remnants of the notochord, accounting for 1%–4% of all primary bone tumors [1]. They most commonly arise along the axial skeleton, particularly in the sacrococcygeal region, skull base, and mobile spine [2]. Extra-axial chordomas (EAC) involving the appendicular skeleton are exceedingly rare, with only isolated cases reported in the literature. In one of the largest contemporary reviews, Hoogervorst *et al*. identified 21 reported adult cases of EAC involving the lower extremities, highlighting the exceptional rarity of these tumors in the appendicular skeleton [3]. Prior series and case reports have described EAC arising in the femur, tibia, fibula, and periarticular regions of the knee, often posing significant diagnostic challenges due to their nonspecific clinical and radiographic features and frequent mimicry of benign or low-grade lesions [4–10].

We present a rare case of an EAC involving the distal tibia that was incidentally discovered during operative fixation of a presumed pathologic fracture following a low-energy twisting injury. Unlike most previously reported cases, which presented with prolonged pain or an enlarging mass, this lesion was initially suspected to represent a benign cystic process and was identified only after intraoperative excision. This case adds to the limited literature on appendicular EAC by highlighting an unusual traumatic presentation, the potential for benign radiographic and gross appearance, and the importance of maintaining suspicion for occult malignancy in atypical cortical lesions encountered during orthopedic trauma surgery.

### Statement of informed consent

Verbal informed consent was obtained from the patient for publication of her clinical history,

radiographic images, and surgical details.

## Case report

**Figure 1:**
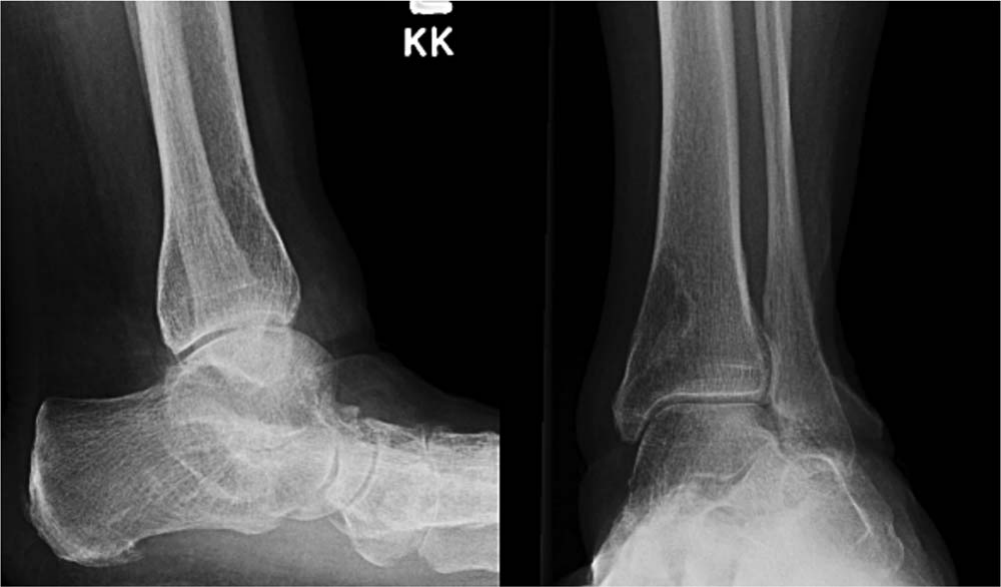
Preoperative anteroposterior and lateral radiographs of the left tibia and fibula demonstrating a spiral fracture of the distal tibial shaft with an associated proximal fibular fracture.

A 59-year-old woman with a medical history significant for rheumatoid arthritis treated with upadacitinib, hypothyroidism, depression, anxiety, and hypertension presented to the Emergency Department following a low-energy fall while walking, resulting in immediate left leg pain and inability to ambulate. Plain radiographs demonstrated a closed spiral fracture of the distal tibia with an associated proximal fibular fracture (Fig. 1). The limb was immobilized in a long-leg splint, and operative fixation was planned for the following day.

**Figure 2:**
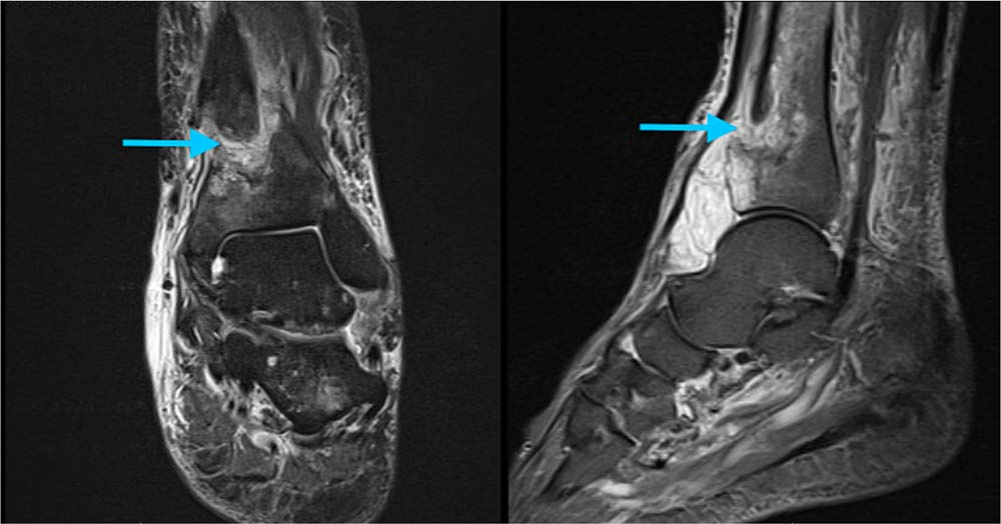
Preoperative MRI of the left ankle. Axial T2-weighted images demonstrate a well-circumscribed, hyperintense soft tissue lesion adjacent to the anterior cortex of the distal tibia (arrow).

During the preoperative evaluation, computed tomography demonstrated erosive changes of the anterior tibial cortex with associated swelling of the anterior compartment. On further history, the patient reported several weeks of painless anterior ankle swelling prior to the injury and denied constitutional symptoms. These findings raised concern for a pathologic fracture, prompting further evaluation with magnetic resonance imaging prior to operative intervention (MRI). MRI demonstrated a well-circumscribed soft tissue lesion with high T2-weighted signal intensity adjacent to the anterior distal tibial cortex (Fig. 2). Based on these findings, operative fixation with concurrent lesion excision was planned.

An anterior approach to the ankle was undertaken along the course of the tibialis anterior tendon. The tendon sheath was longitudinally incised, exposing a well-encapsulated mass consistent with a ganglion cyst abutting the anterior tibial cortex. The lesion was excised in its entirety, including the involved cortical bone, and submitted for histopathological examination. The fracture was anatomically reduced and stabilized using an anteromedial distal tibial plate. Following copious irrigation and layered closure, the patient was transferred to the post-anesthesia care unit in stable condition.

Surgical pathology was evaluated at both our institution and Massachusetts General Hospital. Gross examination revealed a 5.2 × 3.7 × 2.5 cm multiloculated cystic mass with tan-white, fibrotic surfaces, and areas of brown-red gelatinous variegation. Histologic analysis demonstrated cohesive nests of cells embedded within an abundant extracellular myxoid matrix with focal chondroid differentiation, consistent with a conventional chondroma. The lesion was staged as pT1 according to the American Joint Committee on Cancer (AJCC) 8th Edition Staging Manual [11], with no nodal or metastatic category assigned. Immunohistochemistry performed at our institution showed tumor cells positive for S100 and keratin (MNF116). Immunohistochemical analysis at Massachusetts General Hospital demonstrated positivity for brachyury and integrase interactor 1 (INI1). Collectively, these findings confirmed the diagnosis of EAC.

**Figure 3:**
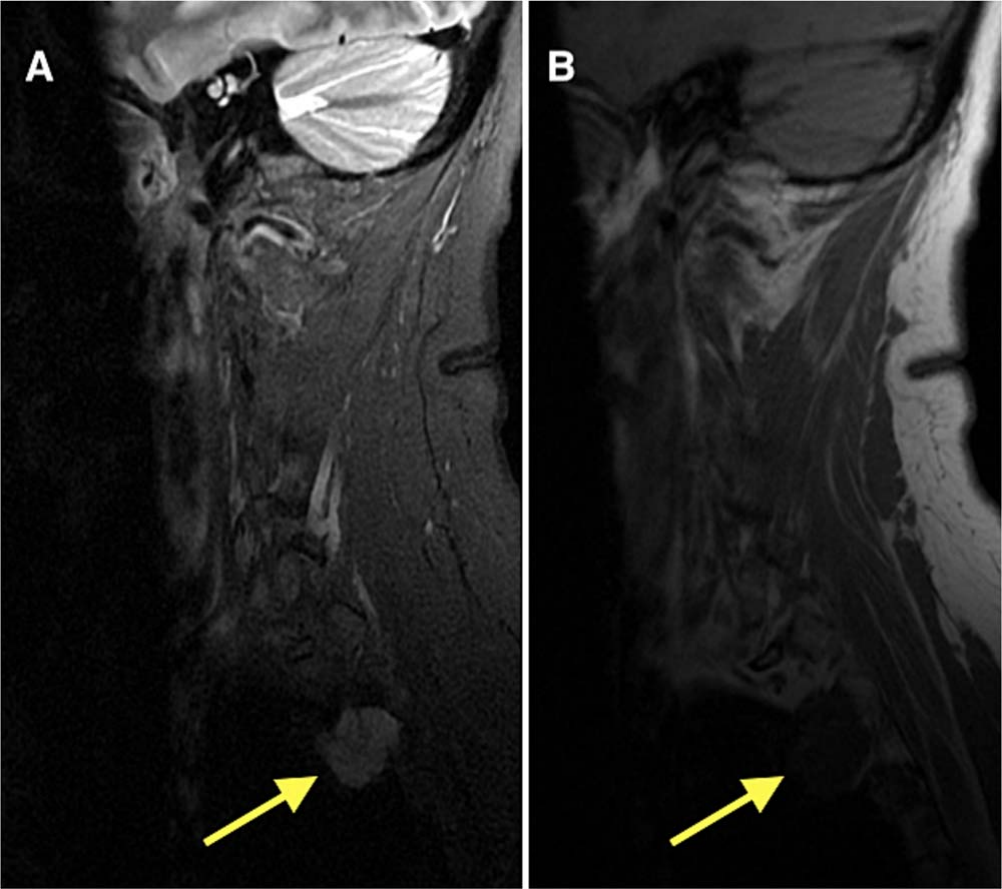
MRI of the thoracic spine demonstrating a lobulated T1-hypointense (B), T2-hyperintense lesion (A) at the lateral margin of the right T2–T3 neural foramen, measuring 2.3 × 2.2 cm (anteroposterior × transverse).

Following diagnosis, the patient was referred to orthopedic oncology and underwent serial multiplanar MRI of the entire spine, as well as CT of the chest. Imaging revealed a lobulated T1-hypointense, T2-hyperintense lesion at the lateral margin of the right T2–T3 neural foramen measuring 2.3 cm (anteroposterior) × 2.2 cm (Fig. 3). Given concern for a potential primary lesion, this mass was subsequently biopsied. The patient continues to follow closely with the oncology service for further evaluation, surveillance, and management.

**Figure 4:**
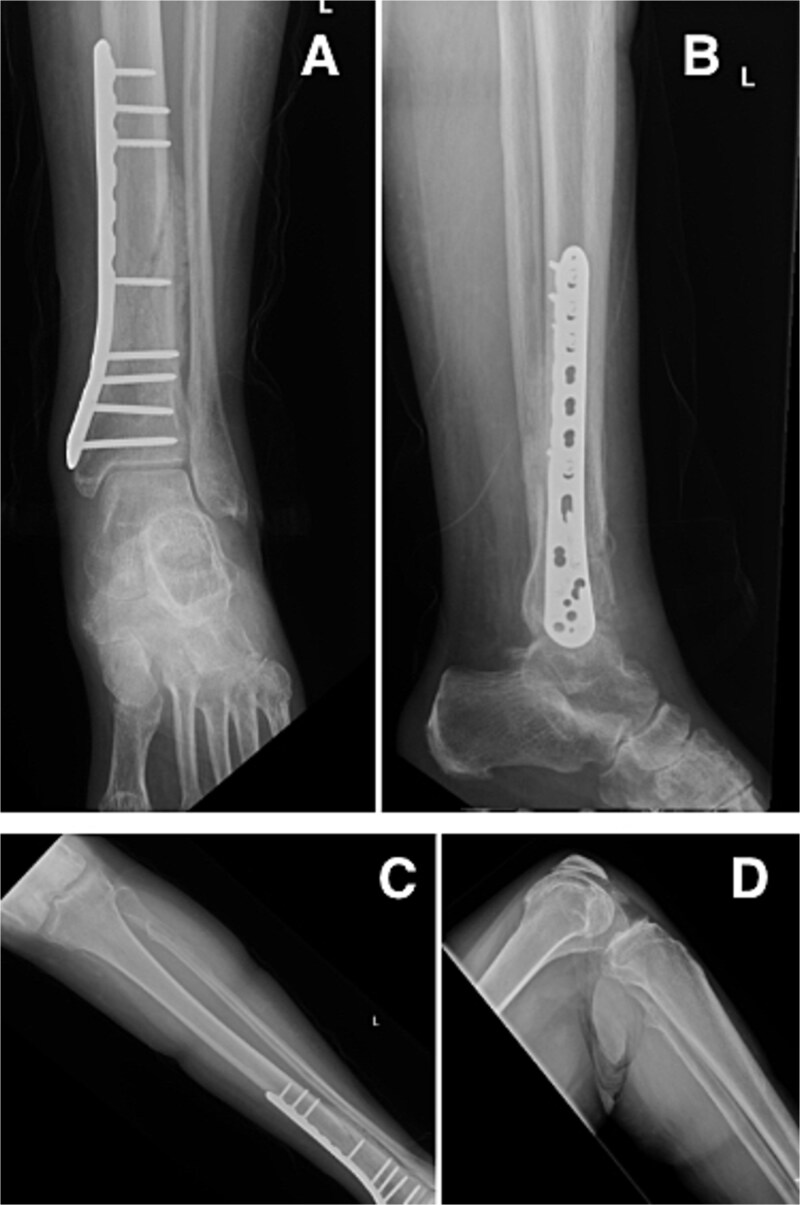
Radiographs at 3-month follow-up demonstrating healing of the left distal tibial spiral fracture and associated proximal fibular fracture. (A) Anteroposterior and (B) lateral views of the distal tibia show maintained alignment and progressive osseous union. (C) Anteroposterior and (D) lateral views of the proximal fibula demonstrate interval fracture healing.

At our latest 3-month follow-up, the patient demonstrated appropriate clinical and radiographic healing of the distal tibial fracture without complication. Interval radiographs confirmed maintained alignment and progressive osseous union at the fracture site (Fig. 4).

## Discussion

The main finding of this case is the identification of an EAC of the distal tibia that was incidentally detected with computed tomography following a low-energy twisting injury. This presentation highlights the diagnostic challenges associated with EAC and highlights the potential for these rare malignancies to masquerade as benign lesions in the setting of orthopedic trauma.

EAC of the appendicular skeleton are exceptionally uncommon, with the largest narrative review by Hoogervorst *et al*. [3] identifying 21 adult cases involving the lower extremities. Within this limited cohort, most patients presented with insidious pain or a slowly enlarging mass, often over several months or years. In contrast, the current case is notable for its acute traumatic presentation, with antecedent painless swelling that was initially clinically silent. Similar tibial cases reported by O’Donnell *et al*., Rekhi *et al*., and Huang *et al*. [4–6] described diagnostic delays or initial concern for alternative pathologies, emphasizing the nonspecific nature of clinical presentation in EAC.

Radiographically, EAC lack pathognomonic features and frequently overlap with benign or low-grade entities such as ganglion cysts, periosteal chondroma, chondromyxoid fibroma, or chondrosarcoma [3]. Prior reports have described cortical erosion with an associated soft tissue component and high T2-weighted signal intensity, features that were also present in this case. As noted by Tirabosco *et al*. and Lantos *et al*. [7, 8], these imaging characteristics often preclude definitive preoperative diagnosis and reinforce the importance of histopathologic evaluation. In our case, the benign-appearing gross morphology further contributed to the diagnostic challenge, as the lesion closely resembled a ganglion cyst intraoperatively.

Histopathologic confirmation remains the gold standard for diagnosis of EAC, with nuclear brachyury expression serving as a highly specific marker, as demonstrated by Tirabosco *et al*. [7]. Brachyury, a transcription factor critical to notochord development, has been shown to be consistently expressed in both axial and EAC and is rarely expressed in histologic mimics, reinforcing its diagnostic utility. Moreover, genomic alterations involving the T (brachyury) gene have been implicated in chordoma pathogenesis, supporting the biological plausibility of chordoma arising outside the axial skeleton despite the absence of native notochordal remnants [12]. Additional immunohistochemical findings further supported the diagnosis. Retained nuclear expression of SMARCB1/INI1 was observed in this case, a feature characteristic of conventional chordoma and helpful in excluding poorly differentiated chordoma, which has been shown to uniformly lack INI1 expression and to exhibit more aggressive behavior [13]. Tumor cell positivity for S100 protein was also identified. While S100 expression is frequently observed in chordomas, it is known to be variable and may be lost in metastatic or recurrent disease [14].

From a management standpoint, wide surgical excision with negative margins remains the cornerstone of treatment for EAC, consistent with prior reports by Evans *et al*. and Righi *et al*. [9, 10]. However, in the trauma setting, definitive oncologic resection may not be feasible at the initial presentation. The present case illustrates a scenario in which stabilization and lesion excision occurred concurrently, with subsequent multidisciplinary oncologic evaluation guiding further management. Chordomas demonstrate a recognized potential for metastatic spread, most commonly to the lung, followed by bone and soft tissue, with metastases to distal bone associated with particularly poor prognosis [15].

EAC of the distal tibia is an exceedingly rare entity that may present with nonspecific symptoms or be discovered incidentally following trauma. This case emphasizes the need for heightened awareness of occult malignancy in atypical cortical lesions encountered during orthopedic trauma surgery, and the importance of follow-up surveillance and management.
